# Management of postintubation tracheal stenosis with bronchoscope methods—An experience from two centers

**DOI:** 10.1002/rcr2.70014

**Published:** 2024-09-08

**Authors:** Arvindran Alaga, Vineet Simhan, Srivatsa Lokeshwaran, Sunil Kumar K, Sanjana Chetana Shanmukhappa

**Affiliations:** ^1^ Pulmonology Department Hospital Sultanah Bahiyah Alor Setar Malaysia; ^2^ Aster Whitefield Hospital Bangalore India; ^3^ Pulmonology Department Aster Whitefield Hospital Bangalore India; ^4^ Pulmonology Department Aster CMI Hospital Bangalore India; ^5^ Internal Medicine Department Unity Hospital Rochester New York USA

**Keywords:** airway stents, balloon dilatation, critical airway stenosis (benign), post intubation tracheal stenosis

## Abstract

Tracheal stenosis is a common complication of endotracheal intubation or tracheostomy, resulting in significant morbidity and mortality. Bronchoscope interventions have been proposed as a safe alternative for the management of post‐intubation post‐intubation tracheal stenosis (PITS). Data for patients diagnosed with PITS across two hospitals, between 2021 and 2022, encompassing demographic, clinical, and procedural details were gathered from electronic medical records, and analysed. Primary outcomes centred on assessing the incidence and severity of PITS through bronchoscope examination and radiological imaging, and the efficacy of bronchoscope interventions, including stenting and the application of mitomycin C. Twelve patients were managed for PITS. Majority of patients were females (9/12) with mean age of 46.41 years. Presenting signs and symptoms were dyspnea, rhonchi and failed extubation, the mean duration of intubation/ tracheostomy is 16.41 days (range: 3–40 days). Most common comorbidity was type 2 diabetes, (5 patients, 41.6%). The lesions mean length was 3.09 cm and Cotton‐Meyer Grade II and III. Prompt evaluation is crucial, in these patients. The Cotton‐Meyer grade is pivotal in treatment decisions, with intubating times correlating with the severity of stenotic disease. Our case series demonstrates the increasing utility of bronchoscopy in managing these cases.

## Graphical abstract

We highlight the pivotal role of cotton‐myer grade in treatment decisions, with intubating times correlating with the severity of stenotic disease. Our case series demonstrates the increasing utility of bronchoscopy in managing these cases.

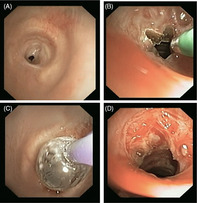

## INTRODUCTION

Tracheal stenosis (TS) arises from various causes, including surgical trauma, intubation, inhalation injuries, or inflammatory conditions. It poses a serious risk to patients who have been intubated or undergone tracheostomy for extended periods. Incidence among intubated patients varies from 10% to 22%, with only 1%–2% experiencing symptomatic or severe stenosis. In the general population, the estimated yearly incidence stands at 4.9 cases per million.^1^ Symptoms may not manifest until weeks or months post‐extubation, potentially leading to misdiagnosis as asthma, with presentations of dyspnea and stridor. Interventional pulmonologists have increasingly played a crucial role in treatment, with bronchoscopic interventions gaining traction in recent years alongside the historically primary approach of tracheal resection with anastomosis. These bronchoscopic techniques, which may include balloon dilatation, radial incisions, or cryotherapy among others, are often followed by subsequent interventions such as stent placement, mitomycin C application, or steroid administration. These additional measures are tailored to the type and severity of stenosis, aiming to enhance success rates. Our case series underscores the efficacy, complication rates, and likelihood of restenosis associated with bronchoscopic interventions for post‐intubation TS.

## MATERIALS AND METHODS

This retrospective case series investigated patients diagnosed with Post‐intubation TS across two hospitals (Aster Hospitals, Bangalore, Karnataka, India and Hospital Sultanah Bahiyah, Alor Setar, Malaysia) between 2021 and 2022. Patient selection involved individuals displaying symptoms consistent with tracheal stenosis post‐intubation, confirmed through clinical examination, imaging studies, and bronchoscopic evaluation, with exclusion criteria applied to those with airway pathologies unrelated to intubation. Data encompassing demographic, clinical, and procedural details were gathered from electronic medical records, including age, sex, comorbidities, intubation duration and surgical interventions, and analysed using Microsoft Excel. Primary outcomes centred on assessing the incidence and severity of tracheal stenosis through bronchoscopic examination and radiological imaging, as well as the efficacy of bronchoscopic interventions, including stenting and the application of mitomycin C. Patients were classified according to Cotton‐Myer grade to evaluate the significance of stenosis severity.^2^ Ethical considerations ensured informed consent was obtained from participants or their legal representatives. Statistical analysis employed descriptive statistics, with continuous variables presented as means and categorical variables as percentages. Data interpretation aimed to elucidate the association between prolonged mechanical ventilation and post intubation TS development, alongside evaluating treatment effectiveness.

## RESULTS

Total 12 patients were managed in both centers for post‐intubation TS. Majority of patients referred to us were females (9/12) with mean age patients of 46.41 years. Presenting signs and symptoms were dyspnea, rhonchi and failed extubation, the mean duration of intubation/tracheostomy is 16.41 days (with range of 3–40 days). Most common comorbidity was type 2 diabetes, seen in 5 patients (41.6%). The lesions mean length is 3.09 cm and they are within the range of Cotton–Meyer Grade II to III. Patient characteristics have been summarized in Table 1.

**TABLE 1:** Patients characteristics.

Age	Sex	Duration of intubation (days)	Tracheostomy	Risk factors	Bronchoscopy (stenosis distance from vocal cords)	Imaging	Cotton–Myer grade
55	F	3	No	DKA	5 cm	0.5 × 0.5 cm	2
71	F	14	No	Endometrial CA	5 cm	0.7 × 0.7 cm	3
33	F	40	Yes	DKA	2 cm	0.5 × 0.3 cm	2
34	F	4	Yes	Asthma	3 cm	0.27 × 0.32 cm	2
52	F	29	No	DKA	6 cm	0.5 × 0.8 cm	3
55	F	7	No	APO	4 cm	0.5 × 0.5 cm	3
53	M	7	Yes	IHD s/p PTCA/DM/HTN	6 cm	0.46 × 0.46 cm	2
37	F	21	Yes	CHD	3 cm	0.3 × 0.6 cm	3
36	M	9	Yes	None	5 cm	0.5 × 0.5 cm	2
35	M	30	Yes	CVA/IHD/ s/p MVR	5 cm	0.86 × 0.4 cm	2
48	F	8	No	DKA	1 cm from carina	0.3 × 0.4 cm	3
48	F	25	Yes	Post COVID /hypothyroid	3 cm	1 × 0.5 cm	2

**FIGURE 1:**
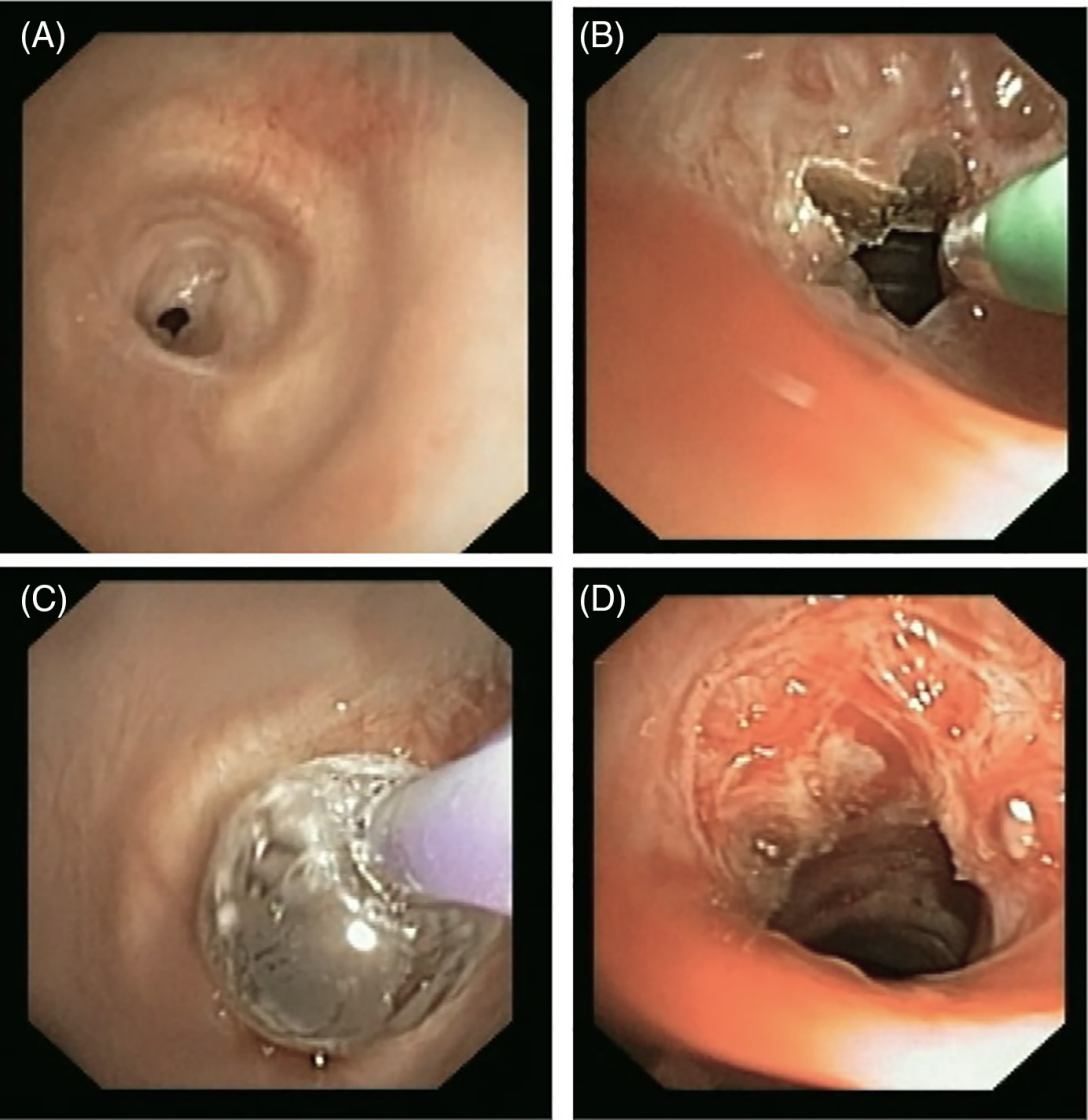
Bronchoscopic view showing the course of patient‐9 (A) With simple tracheal stenosis, (B) Using electrocautery (showing Benz sign cuts), (C) Ballooning to achieve dilation of tracheal lumen, and (D) Trachea post CRE balloon dilation establishing airway.

**FIGURE 2:**
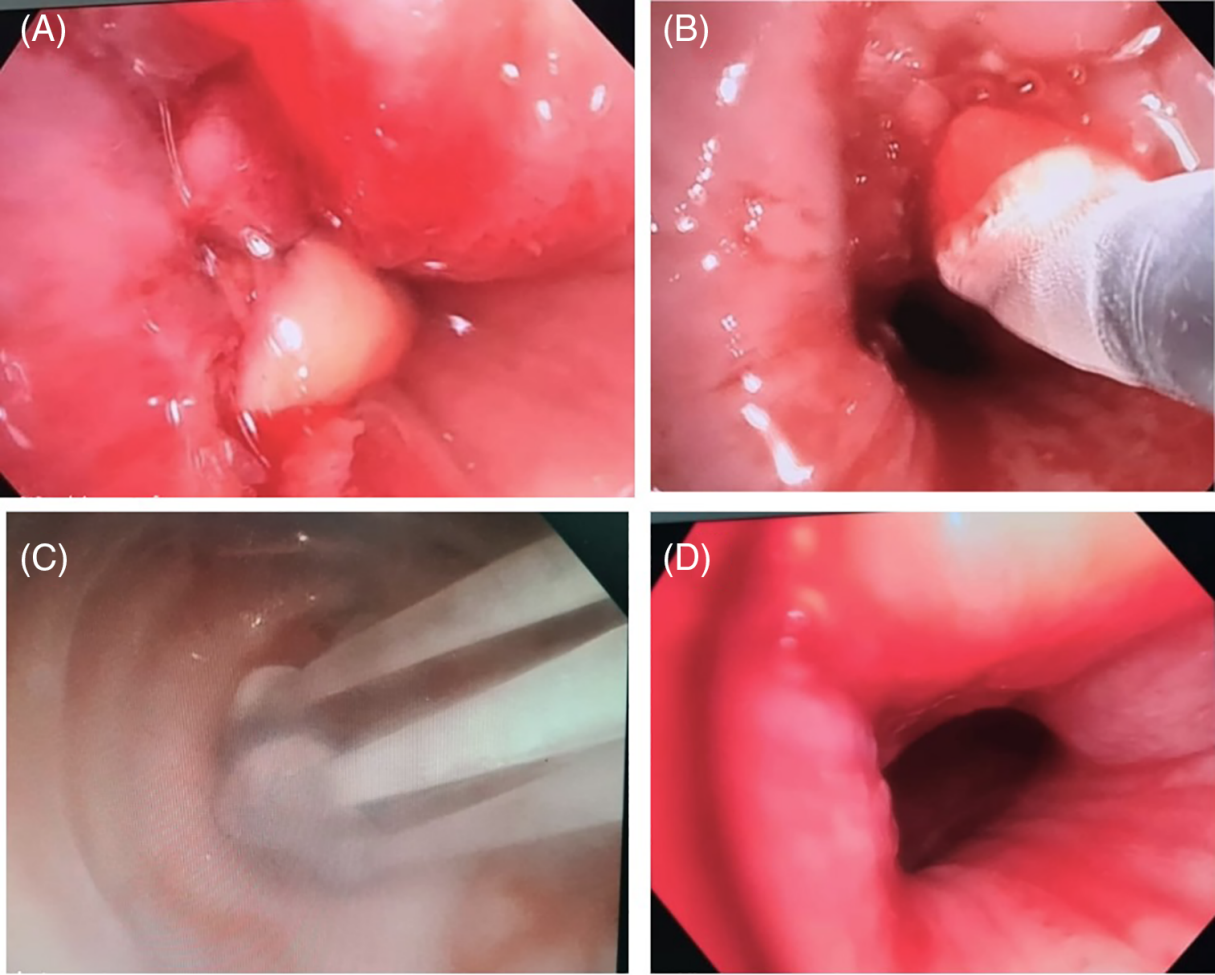
Bronchoscopic view of patient‐11 (A) showing critical tracheal stenosis, (B) Using electrocautery for debulking, (C) Trachealator for establishing an airway lumen, and (D) Patent airway established following debulking.

**FIGURE 3:**
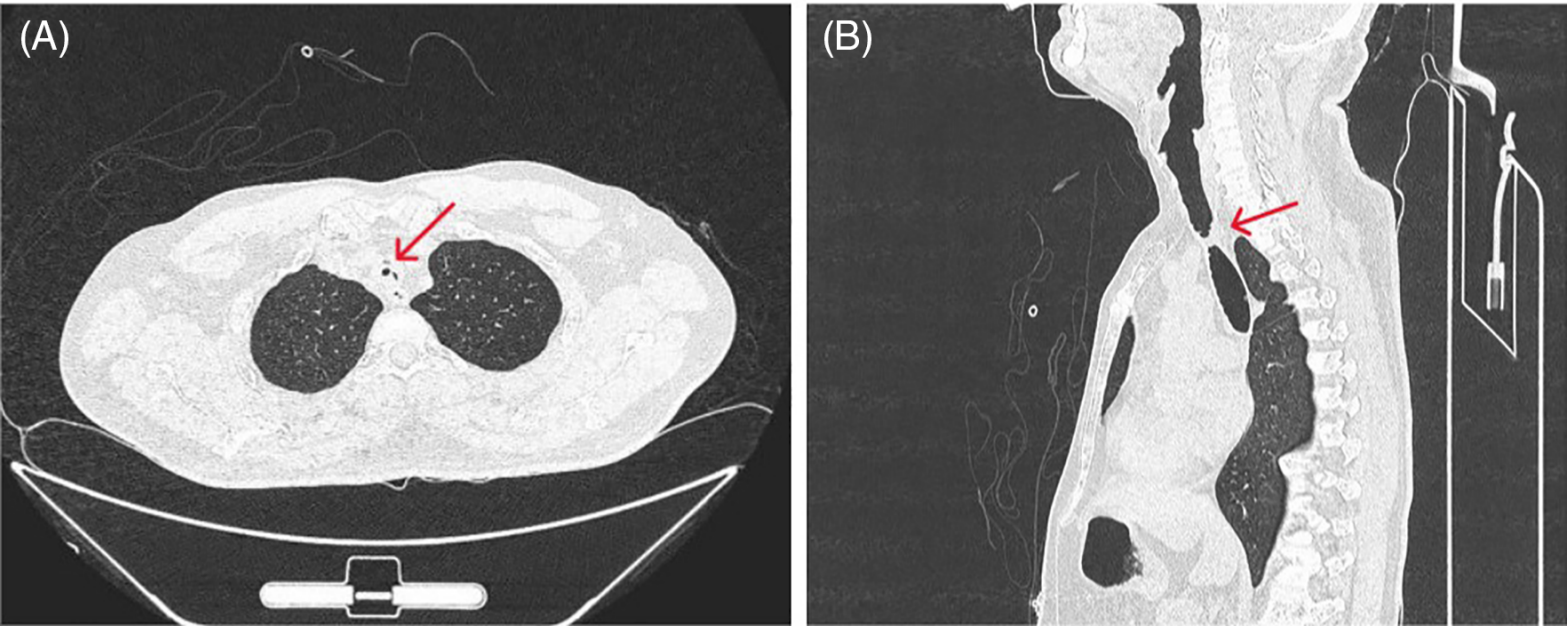
Computed tomography scan of patient‐5 showing complex tracheal stenosis (red arrow) in (A) axial view and (B) sagittal view.

All of them underwent rigid bronchoscopy and mechanical dilatations. Six patients underwent electrocautery cut and triamcinolone injection. Mitomycin c was used in four patients. Five of them needed stent insertion (1 silicone stent and 4 Self expandable metallic stents). One of them underwent the procedures three times to be relieved from the symptoms, 2 patients needed procedure twice and another 1 case were referred for surgical management while other patients were asymptomatic after undergoing the procedure only once. No flow volume loop done for our patients as they present in severe distress symptoms to our emergency services. performed for the patients as they present in No complications like pneumothorax or bleeding post procedure occurred. We noticed middle age female, with underlying diabetes mellitus who were intubated more than 2 weeks highly susceptible to develop post‐intubation TS. Table 2 summarizes the interventions done in the above patients (Figures 1, 2, 3).

**TABLE 2:** Interventions done in patients.

Patient	Rigid bronchoscopy	No. of attempts	Mitomycin c	Stenting	Duration of Stent placement
1	Dilatation x2	1	No	No	
2	Balloon dilatation	1	No	No	
3	Balloon dilatation+ stenting	1	Yes	Yes	3 months 10 days
4	Balloon dilatation	1	Yes	No	
5	Balloon dilatation	2	Yes	No	
6	Balloon dilatation	1	Yes	No	
7	electrocautery cut/ triamcinolone injection/dilatation+ fully covered SEMS	1	No	Yes	4 months 6 days
8	electrocautery cut/triamcinolone injection/dilatation+ fully covered SEMS	1	No	Yes	4 months
9	electrocautery/triamcinolone injection and dilatation	1	No	No	
10	electrocautery cut/triamcinolone injection/dilatation + fully covered SEMS	3	No	Yes	3 months 15 days
11	electrocautery cut/triamcinolone injection/dilatation	1	No	No	
12	electrocautery cut/triamcinolone injection/dilatation+ fully covered SEMS	2	No	Yes	5 months

Abbreviation: SEMS, self expanding metallic stent

## DISCUSSION

Tracheal stenosis can occur after either endotracheal intubation or tracheostomy.^3^ The subglottic region is the most commonly involved (over 90% occurring following prolonged intubation) due to the signet ring anatomy of the cricoid cartilage preventing outward expansion in progressing edema.^1^, ^4^ Pathology is due to pressure necrosis and erosive chondritis at the level of the cuff, with mucosal damage being seen when the cuff pressure exceeds 30 mm Hg.^5^ To reduce this incidence, the use of low‐pressure high‐volume cuffs have seen widespread use recently.

Most common co‐morbids include diabetes (impaired fibroblast migrations) and obesity (reduced thoracic compliance requires higher ventilatory pressures).^6^ Increased incidence of post‐intubation TS in women has been linked to oestrogen‐induced elevation of transforming growth factors, deposition of type I and III collagen, leading to fibrosis.^1^ The surge in post‐intubation TS incidence during the COVID‐19 pandemic, attributed to prolonged ventilation, is exacerbated by the theory that repeated pronation cycles may increase tube pressure at the anterior or posterior glottic region, heightening the risk.^6^ Many of these COVID‐19 patients with post‐intubation TS presented with delayed symptoms after ICU discharge, due to which targeted evaluation at ruling out post‐intubation TS is recommended.

Clinical symptoms (dyspnea, cough, wheezing, stridor, and recurrent infections) may be quite delayed due to which it is commonly misdiagnosed as asthma.^7^ Initial investigation always includes fibre‐optic bronchoscopy as a first line which can show the presence, type, location and other characteristics of the stenosis (mucosal inflammation and number of rings involved and calibre). A neck CT is generally done to check for any tracheal wall involvement.^4^, ^7^

Stenosis can be classified as either simple or complex based on certain features which helps guide management. Simple stenosis is defined as involving less than 1 cm of the trachea, without evidence of malacia or loss of cartilage, while complex stenosis involves more than 1 cm with varying degrees of cartilaginous involvement.^8^ Complex stenosis is more commonly seen in previously tracheostomized patients. Post‐intubation TS management is complex and most patients need more than one procedure to achieve stabilization.^1^, ^9^ A study done Beomsu Shin et al compared the clinical presentation and outcomes between patients with post intubation versus post tracheostomy.^10^ Post tracheostomy patients were found to have more complex clinical courses, longer ventilator dependence and longer hospital stays compared to post intubations post‐intubation TS cases. Severe and mixed type stenosis were also more commonly seen in post‐tracheostomy TS patients.

Simple post‐intubation TS is generally treated with endoscopic balloon dilation. Stenosis caused by granulation tissue can be treated with the help of Nd: YAG laser or electrocautery, while web‐like stenosis is generally treated with mucosal sparing technique with the help of radial incisions.^1^, ^9^ Cryotherapy has recently been proposed as a novel treatment for cases of post‐intubation TS. It helps in accelerating the healing response, resulting in less fibrosis as the integrity of the extracellular matrix is left intact. Further studies are still needed to validate the efficacy of cryotherapy to treat post‐intubation TS.^5^

Surgical resection along with end‐to‐end anastomosis have been considered the gold standard treatment for complex post‐intubation TS. This method has a few drawbacks, including restenosis and regrowth of granulation tissue at the site of the anastomosis.^11^, ^12^ In recent times, bronchoscopic interventions have played an important role in acting as a bridge to surgery, while also being considered as definitive treatment in patients unable to tolerate surgery.^10^, ^11^ Endoscopic application of steroids or mitomycin c has been used by some surgeons to reduce scar formation and the risk of restenosis.^3^ Endoscopic treatment offers minimal morbidity with good functional outcomes; however, stenosis can recur and repeated dilations may be required. Surgery still remains the treatment of choice if bronchoscopic measures fail. Complex post‐intubation TS can also be treated with serial dilations. As the number of dilations increases so does the time until the next dilation, demonstrating stenosis stabilization along serial interventions.^9^ We opted for covered Self‐Expanding Metallic Stents (SEMS) primarily for their ease of insertion and removal, a crucial requirement for the majority of our patients. Moreover, given the elevated risk of migration associated with silicone stents, we favoured the utilization of SEMS stents.^12^ Additionally, most of our patients presented to us in a state of emergency due to which we deployed readily available SEMS to bring rapid relief of symptoms.

Two studies by Mustafa and Freitas et al. reviewed 152 post‐intubation TS patients who underwent either tracheal resection with end‐to‐end anastomosis or bronchoscopy. Success rates with tracheal resection were 64.9% with common complications including restenosis and anastomotic dehiscence.^13^ In the second study, simple post‐intubation TS were managed with bronchoscopic interventions, while complex cases required surgery (30%) or stent placement (19%). Post‐surgical recurrences were managed by mechanical dilations, repeat surgery (1 case), or silicone stent placement; 5 patients with complex post‐intubation TS underwent tracheostomy.^9^ Combining data obtained from several articles, Table 3 highlights and compares the success, restenosis and complication rates between surgical and bronchoscopic modalities for first line treatment of post‐intubation TS.

**TABLE 3:** Success, restenosis, and complication rates between surgical and bronchoscope modalities for first line treatment of post‐intubation tracheal stenosis.

	Surgery^3^, ^13^, ^14^	Bronchoscopy with dilation^7^, ^8^, ^11^
Success rate	70/84 (83.3%)	189/218 (86.6)
Restenosis rate	9/84 (10.7%)	21/218 (9.6%)
Complications	34/84 (40.4%)	10/44 (22.7%)

Our two‐year case series, conducted across 2 centers in Malaysia and India, demonstrates that bronchoscopic interventions, with or without stent placement, are a safe alternative to traditional surgery for post‐intubation tracheal stenosis. Unlike other studies that compare treatment modalities based on stenosis complexity,^2^, ^6^ our series focuses on a diverse South‐East Asian patient population. We identified potential risk factors in the Asian population, including diabetes, heart failure, and a higher incidence in women. The study emphasizes a multimodality approach, such as using a trachealator, mitomycin C, and triamcinolone injection, tailored to patient profiles for optimal outcomes. It underscores the importance of rapid bronchoscopic therapies for emergencies and highlights the favourable outcomes of self‐expanding metallic stents (SEMS) in critical situations. Our findings suggest that bronchoscopic interventions should be considered first‐line therapy, especially for patients presenting in emergencies.

Limitations of our study included its retrospective design, potential selection bias, and reliance on data from a limited number of institutions, possibly constraining the generalizability of findings.

In conclusion, prompt evaluation for post‐intubation TS is crucial, particularly in patients with a history of prolonged intubation or tracheostomy, presenting with dyspnea, rhonchi, and failed extubation. Following the COVID‐19 pandemic, there has been a notable increase post‐intubation TS cases, emphasizing the urgent need for recognition and improved treatment strategies. Our study highlights the pivotal role of the Cotton‐Myer grade in treatment decisions, with intubation times correlating with the severity of stenosis. While simple cases often respond well to bronchoscopic interventions, complex stenosis typically requires surgical or stent‐based approaches, despite associated risks such as restenosis, as observed in our study. Our case series demonstrates the increasing utility of bronchoscopic interventions as a safe alternative, with or without stent placement and mitomycin C application, in managing these cases, thereby avoiding unnecessary surgeries. Further research into optimizing mitomycin C application and stent placement is warranted to ensure their efficacy before widespread implementation.

## AUTHOR CONTRIBUTIONS

Arvindran Alaga, Srivatsa Lokeshwaran, and Vineet Simhan were involved in the concept and design of the manuscript. Arvindran Alaga, Srivatsa Lokeshwaran, Vineet Simhan, Sunil K. Kumar, and Sanjana Chetana Shanmukhappa were involved in the critical review and data analysis. Arvindran Alaga provided overall supervision. All authors reviewed and approved the final version of the manuscript.

## CONFLICT OF INTEREST STATEMENT

None declared.

## ETHICS STATEMENT

The authors declare that appropriate written informed consent was obtained for the publication of this manuscript and accompanying images.

## Data Availability

The data that support the findings of this study are available on request from the corresponding author. The data are not publicly available due to privacy or ethical restrictions.
